# Structural engineering of flagellin as vaccine adjuvant: quest for the minimal domain of flagellin for TLR5 activation

**DOI:** 10.1007/s11033-024-10146-y

**Published:** 2025-01-07

**Authors:** Haroon Afzal, Asad Murtaza, Li-Ting Cheng

**Affiliations:** 1 International Degree Program of Animal Vaccine Technology, International College, National Pingtung University of Science and Technology, 1, Shuefu Road, Neipu, Pingtung 91201 Taiwan; 2 Norwegian College of Fishery Science, Faculty of Biosciences, Fisheries and Economics, UiT - The Arctic University of Norway, Tromsø, Norway; 3 Graduate Institute of Animal Vaccine Technology, College of Veterinary Medicine, National Pingtung University of Science and Technology, 1, Shuefu Road, Neipu, Pingtung 91201 Taiwan

**Keywords:** Flagellin, TLR5, Adjuvant, Hapten, Dimerization

## Abstract

Flagellin stimulates Toll-like receptor 5 (TLR5), triggering both innate and adaptive immune responses, making it a potential vaccine adjuvant. On mucosal surfaces, flagellin induces a strong release of cytokines, chemokines, and immunoglobulins. When used in its free monomeric form, flagellin has been shown to enhance immune responses when combined with vaccine antigens. Further research demonstrated that genetically linking flagellin to the antigen provides a more consistent immune boost. However, the bulky structure of flagellin presents challenges in designing the antigen-adjuvant construct, leading to ongoing research to determine the minimal flagellin domain necessary for its adjuvant effect. Early findings suggest that only the D0 and D1 domains are required for immune enhancement. Functional analysis revealed that the TLR5-binding region is located in the D1 domain, while TLR5 dimerization and signaling require the presence of D0. Further reductions in the size of the D0 and D1 domains may be possible as deeper studies aim to identify the key residues responsible for TLR5 activation and immune enhancement. Additionally, flagellin is being tested as a hapten carrier alongside its established adjuvant role. Recently, significant advancements in flagellin application have been observed as it progresses through clinical studies as an adjuvant, anti-radiation, and anti-cancer agent.

## Flagellin activates toll-like receptor 5, leading to innate and adaptive immunity, and may serve as a vaccine adjuvant

*Flagellin is one of the Pathogen-Associated Molecular Patterns (PAMPs).* Flagellin is the major structural protein of the bacterial flagellum, a whip-like extracellular structure responsible for the locomotion of flagellated bacteria [1, 2]. In addition to providing motility, the bacterial flagellum is also involved in adhesion, biofilm formation, post-infection dispersal, and immune modulation, making it an indispensable structure for bacteria survival. The bacterial flagellum consists of three parts: the basal body (membrane-anchored reversible motor that powers the rotation of the filament), the hook (protruding from the basal body to act as a universal joint), and the filament (extending from the hook, a long whip-like propeller composed of flagellin monomers) [3]. Since the flagellum is an important and evolutionarily conserved structure, the presence of its major component, the flagellin monomer, has become a sign of bacterial infection for the host immune system. Flagellin, along with other molecular structures commonly associated with pathogens, are termed Pathogen-Associated Molecular Patterns (PAMPs) [4]. Hayashi et al. were among the few people who demonstrated flagellin recognition by the TLR5 in earlier days. The group elaborated on the activation of NF-kB and the release of cytokines. They performed gene knockout method studies and verified that the TLR5 activation changes dramatically by the presence and deletion of the flagellin gene [5].

## The immune system recognizes PAMPs as signs of infection through toll-like receptors (TLRs)

To detect the invasion of pathogens and initiate protective responses, the innate immune system displays a variety of pattern recognition receptors (PRRs), including (i) the Toll-like receptors (TLRs), (ii) the Retinoic acid-inducible gene I (RIG-I) -like receptors, (iii) the Nucleotide-binding and oligomerization domain (NOD)-like receptors, and (iv) the C-type lectin receptors [6, 7]. The human TLRs and their cognate ligands (PAMPs) are: TLR1 (lipopeptides), TLR2/6 (lipoprotein), TLR3 (double-strand RNA), TLR4 (lipopolysaccharide), TLR5 (flagellin), TLR7/8 (single-strand RNA), and TLR9 (CpG oligodeoxynucleotides) [8].

## Activated TLRs lead to innate and adaptive immunity

**Fig. 1:**
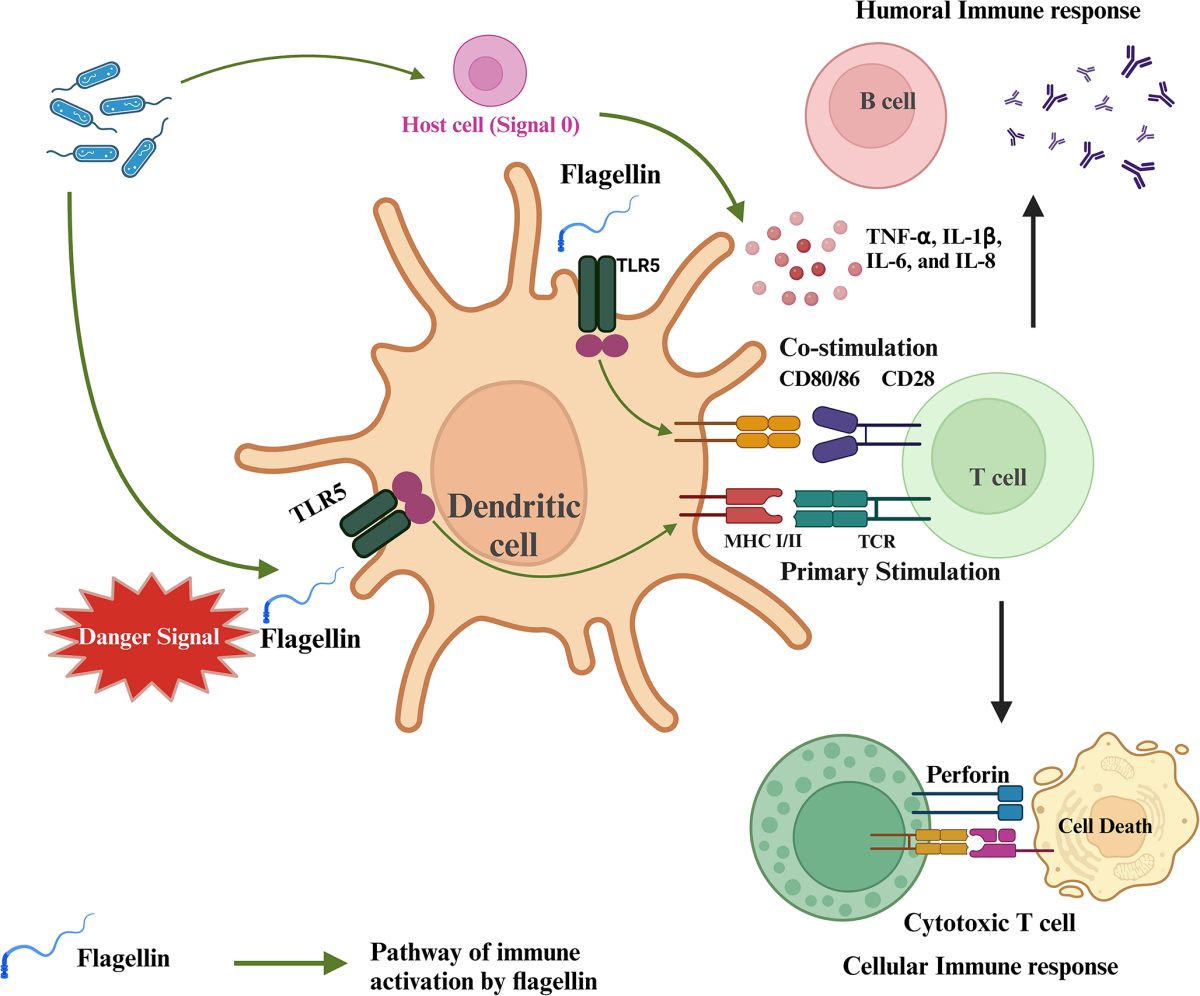
Flagellin acts as a danger signal by activating PRRs on dendritic cells, enhancing antigen presentation and upregulating cytokines and co-stimulatory molecules. This process amplifies both innate and adaptive immunity, ensuring a robust and coordinated immune response. Created in Biorender

When PAMPs bind and activate their cognate TLRs, the innate immune response is initiated at the site of infection [9]. Three groups of cells are involved in the TLR-mediated innate immune response. (1) Epithelial cells at common infection sites such as the intestinal, respiratory, and urogenital tracts express an array of TLRs [10, 11]. (2) Also at the infection site are resident innate leukocytes such as dendritic cells (DCs), macrophages, and mast cells that can be activated through PAMPs to release inflammatory cytokines [12]. Finally, (3) circulating lymphocytes are recruited to the infection site when PAMPs activate TLR-expressing endothelial cells of the blood vessel for immune cell recruitment [13]. Overall, the presence of PAMPs at the infection site activates TLR-expressing immune cells to produce an inflammatory cytokine milieu that leads to pathogen clearance by the innate immune cells [14]. In addition to inducing innate immune response, PAMPs are also critical for facilitating the development of adaptive immunity. At the infection site (peripheral tissues), adaptive immunity is initiated when immature DCs phagocytose microbial antigens for presentation. If PAMPs are also present, TLR-signaling will activate DCs to switch from antigen-collection mode to presentation mode. Stimulation of immature DCs with PAMPs promotes changes in receptor expression that allow DCs to migrate from peripheral tissues to the draining lymph nodes. During this migration, DCs undergo a maturation program for effective antigen presentation to stimulate naïve T cells [15]. When inside the lymph node in the T cell area, DCs can activate naïve antigen-specific T cells by providing them with the two required signals: (1) antigen in the context of major histocompatibility complex (MHC) molecules and (2) costimulatory molecules such as B7-1 (CD80) and B7-2 (CD86). PAMP-mediated TLR signaling is the main pathway by which DCs become activated and mature to express the costimulatory molecules mandatory for T-cell activation. Depending on the costimulatory molecules expressed and the cytokines produced by DCs, primary activation of CD4+ (differentiation into either TH1 or TH2 cells) and CD8 + T cells occurs, leading to adaptive immunity. In summary, PAMPs activate DCs through TLR-signaling to generate antigen-specific adaptive immune response (Fig. 1) [16].

*Because PAMPs serve as the necessary ‘danger signal’ to the immune system*,* PAMPs may be included in modern-day subunit vaccines for enhanced efficacy* [17]. Over the years of vaccine technology advancement, the antigen components of vaccines have moved away from live and inactivated pathogens to synthesized subunit antigens in the interest of safety and ease of scale-up production. The production of only the antigenic parts of pathogens in established heterologous expression systems allows efficient manufacturing of non-infectious antigens [18]. However, the safe nature of these simple components also means that they lack the typical pathogen structures required to signal to the immune system for immune response. In other words, the immune system does not perceive the purified antigens as threats. Therefore, it has become necessary to include macromolecules from pathogens, such as PAMPs, in vaccine formulations to elicit robust immune response [19, 20].

## Flagellin interacting with TLR5 forms a major innate immune response mechanism at mucosal barriers

Mucosal surfaces in the respiratory, urogenital, renal, and intestinal tracts are heavily populated with TLR5 receptors on epithelial cells. These epithelial cells are the first cells to encounter pathogens and their toxins. The presence of TLR5 on mucosal epithelial cells and immune cells, such as dendritic cells and macrophages, underpins the role of flagellin as a mucosal adjuvant [21]. Research on intestinal CD11c + lamina propria cells (LPCs) indicates that flagellin is a vital stimulator of intestinal immunity, even more so than lipopolysaccharides (LPS) [22]. Since LPCs lack TLR4 receptors, TLR5 activation is primarily responsible for the release of chemokines and cytokines (IL-8, IL-12, and IL-6) on mucosal surfaces. Epithelial detection of flagellin is followed by cytokines release which recruit immune cells to mucosal surfaces. These immune cells like dendritic cells, T-cells, and B-cells link the robust innate response to adaptive immunity.

**Fig. 2:**
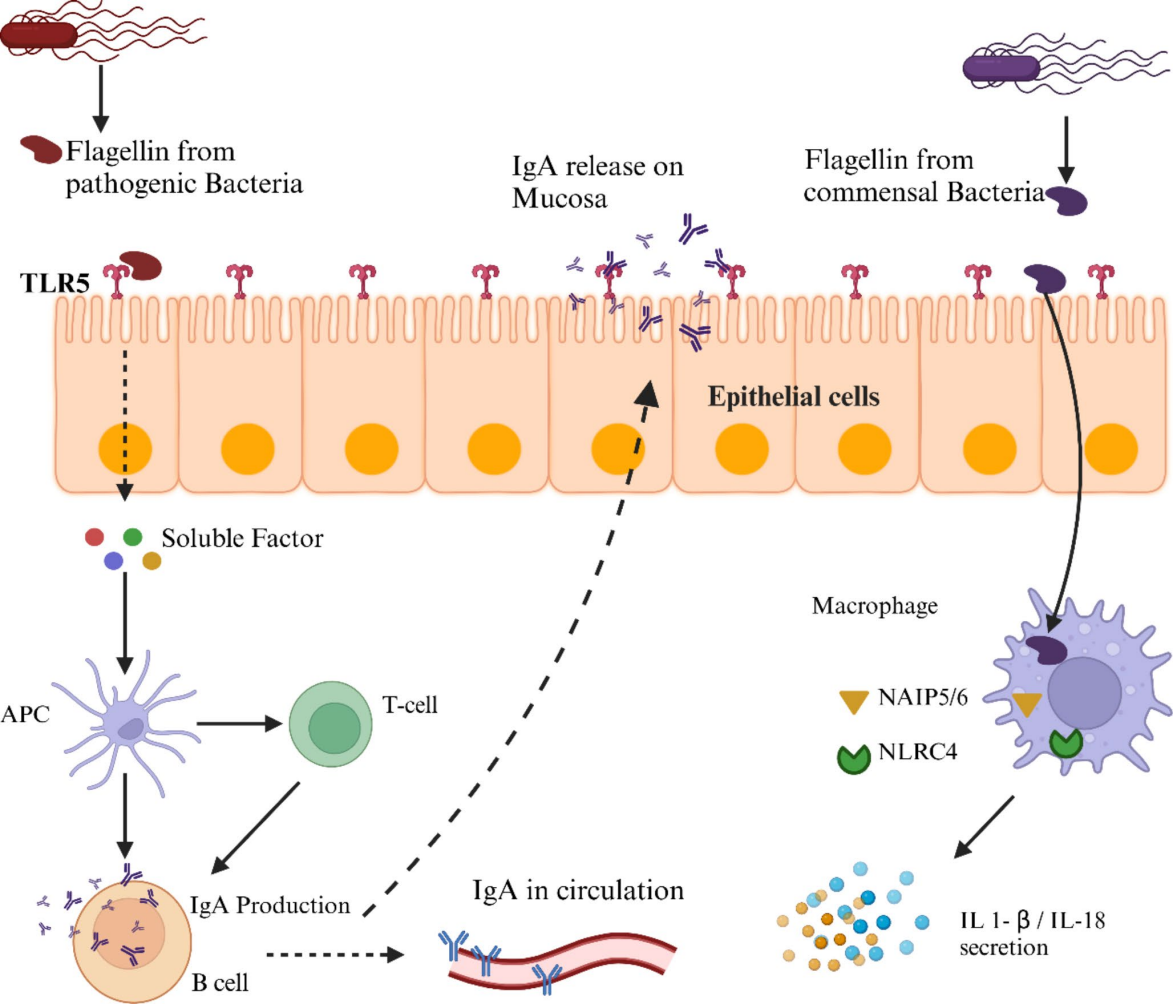
Epithelial cells recognize flagellin through surface TLR5, which leads to the activation of antigen-presenting cells (APCs). These APCs process and present the antigen to T-cells, subsequently activating B-cells. The activated B-cells produce IgA antibodies, which enter the circulation and also migrate to the lumen, where they capture and opsonize pathogens. Created in Biorender

Protective immunity achieved through mucosal vaccination protects in the following ways (i) intensive release of cytokines, and chemokines (ii) nitric oxide on mucosal surfaces, and (iii) release of IgA on mucosal surfaces which control pathogen entry [22]. Mucosal application of flagellin provides robust dual protection by inducing both mucosal (IgA) and systemic (IgG) immune responses. IgA, transported to mucosal surfaces such as the respiratory, gastrointestinal, and urogenital tracts, and into the milk of lactating animals, plays a critical role in newborn protection (Fig. 2). In milk, IgA defends newborns against diseases like porcine epidemic diarrhea virus (PEDV) [23]. Numerous studies have been conducted to explore the potential of flagellin as a mucosal adjuvant. However, flagellin as a protein faces challenges of degradation by proteases and clearance by cilia [24]. These hurdles were overcome by inventing a delivery system that ensures safe and efficacious uptake of flagellin [25]. In the case of the subunit influenza virus vaccine, a short antigen 3M2e was attached to the C-terminus of *B. subtilis* flagellin Hag. Proteinaceous nanoparticles were designed and the flagellin-3M2e complex was displayed on the surface of the nanostructure. Intranasal immunization in mice stimulates antigen uptake, IgA release, and protection against lethal viral challenge [26]. However, the direct application of flagellin also showed suitability for mucosal application. Studies on disease models like *Porphyromonas gingivalis* [27], *Plasmodium falciparum* [28], and *Vibrio vulnificus* [29] supported enhancement in mucosal immune response to flagellin as co-administered or linked to antigen. Alternative routes for mucosal flagellin administration, such as subconjunctival and intraperitoneal, have demonstrated effectiveness in combating *Pseudomonas aeruginosa* infections. Prophylactic application of flagellin has been shown to prevent inflammation and keratitis in murine models [30].

## Structural studies of flagellin-TLR5 interaction allowed better designs of flagellin as an adjuvant

To include flagellin in a subunit vaccine for enhanced immune response, several strategies can be envisioned: (1) simple mixture of flagellin with antigen, (2) genetically linking flagellin to antigen, and (3) identifying only the TLR5-activation domains of flagellin to be linked to antigens. Simply mixing flagellin monomers with vaccine antigen is an intuitive first approach and some immune enhancement can be observed [31]. However, investigators soon started to physically link flagellin to antigens for improved efficacy since the simultaneous presentation of antigens and PAMPs to DCs should efficiently initiate adaptive immunity [32]. Finally, for higher protein expression of the antigen-flagellin fusion construct, efforts have been made to identify the minimal domain of flagellin necessary for TLR5 activation [33].

## X-ray crystallography of flagellin-TLR5 binding

**Fig. 3:**
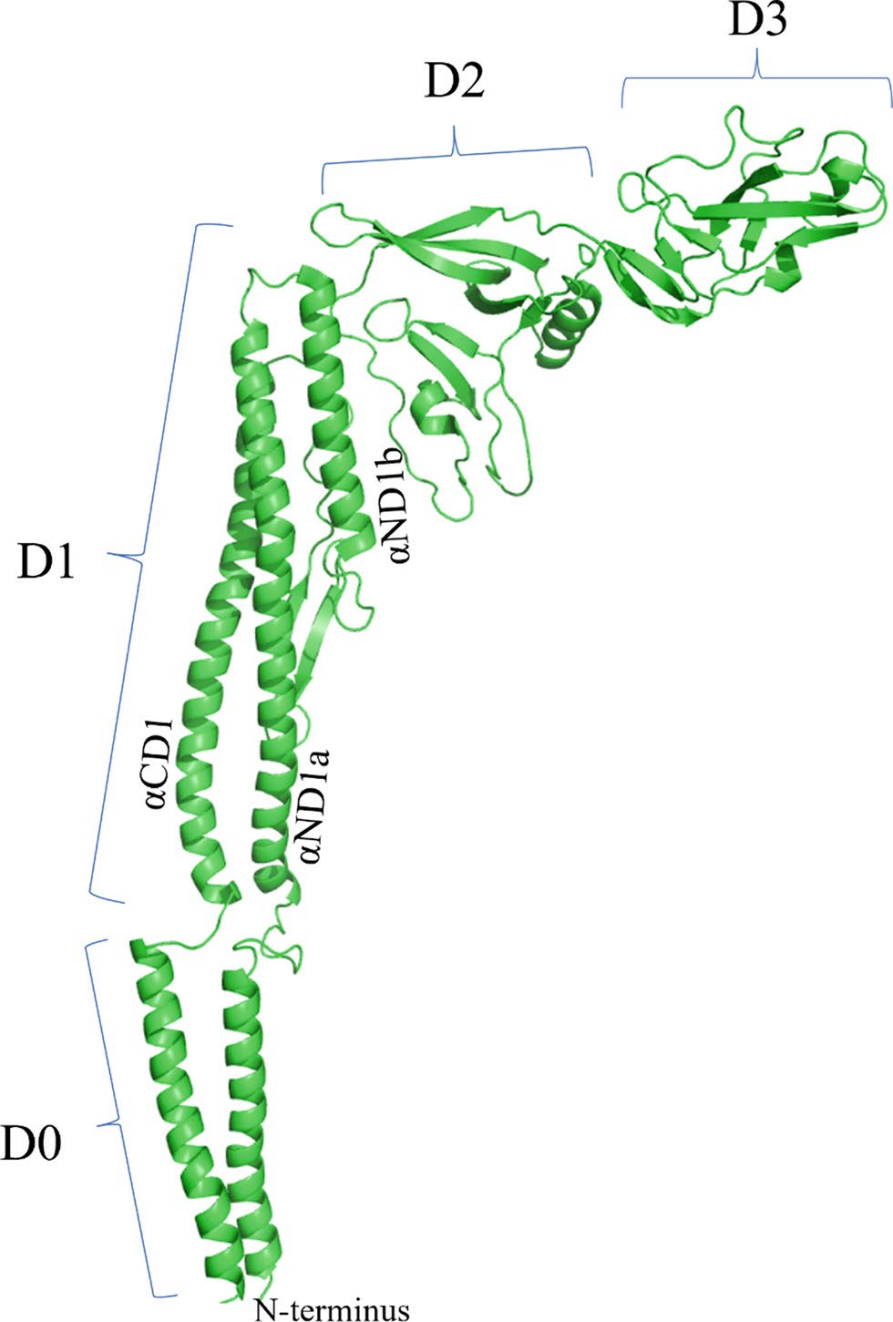
Crystal structure of flagellin. The previously solved 3D structure of the full-length flagellin (Protein Data Bank ID: 1UCU) as illustrated by the PyMol server is shown containing domains D0, D1, D2 and D3

Structural interaction between flagellin and TLR5 has been solved at the atomic level, providing a framework for functional analysis of flagellin [34]. Flagellin from *S. typhimurium* can be separated into four domains, D0, D1, D2, and D3, arranged in a boomerang-like structure [35]. The structure of flagellin was taken from PDB ID 1UCU [34, 36] (Fig. 3).

**Fig. 4:**
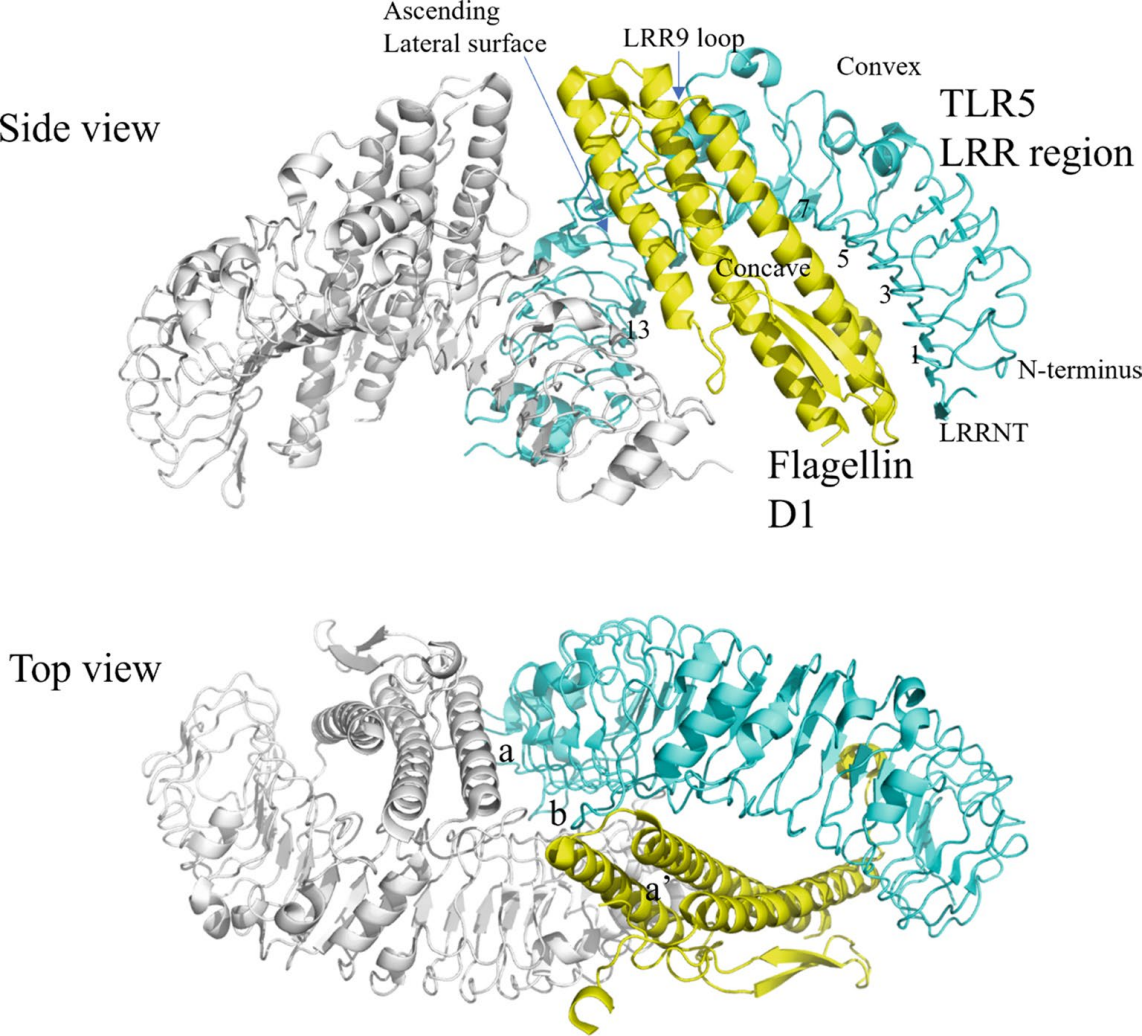
Crystal structure of flagellin D1 and the LRR region of TLR5 in 2:2 complex. Previously solved 3D structure of the zebrafish TLR5 (as a variable lymphocyte receptor hybrid protein) in complex with Salmonella flagellin (Protein Data Bank ID: 3V47) is illustrated by the PyMol server

In terms of protein sequence, the domains of flagellin are arranged as, starting at the N-terminus, D0-D1-D2-D3-D2-D1-D0. When flagellin monomers polymerize to form the flagellum filament, D0 and D1 are embedded within the core of the filament while D2 and D3 protrude from the surface. Comparative analysis of flagellin from different bacteria showed that D0 and D1 are highly conserved whereas D2 and D3 show greater variability in sequence and structure [36]. In actual application, an engineered polypeptide drug (CBLB502) containing complete D0 and D1 was shown to retain full ability to activate NF-kB signaling through TLR5, indicating that D2 and D3 are expendable for signaling [37]. As for the structure of TLRs, it consists of a horseshoe-shaped ectodomain, a transmembrane domain, and an interior part called the Toll/interleukin-1 domain (TIR). The ectodomain contains leucine-rich repeats (LRRs) that serve as the ligand-binding site. The cytoplasmic TIR domain is divided into three conserved boxes, called Box 1, 2, and 3, that vary in size and are critical signaling portions of the molecule; their side chains are used for interaction with downstream signal adaptor molecules [38]. Overall, like other TLR-ligand interactions, the binding of flagellin leads to TLR5 dimerization, bringing the C-terminal regions into juxtaposition so that their intracellular TIR domains can initiate a signaling cascade. For flagellin-induced TLR5 activation, signaling can be MyD88-dependent (or TRIF-mediated pathway and leads to NF-kB activation for expressions of inflammatory cytokines [39]. Notably, flagellin can also be detected intracellularly by another PRR, NAIP5-NLRC4, leading to inflammasome formation and immune activation [40]. When Yoon et al. co-crystalized *Salmonella* flagellin with TLR5 from zebrafish, D1 of flagellin was found to actively engage in TLR5 binding in 1:1 and 2:2 symmetry; that is, two TLR5-Flagellin 1:1 heterodimers assemble into a 2:2 tail-to-tail signaling complex [41] (Fig. 4).

In the solved TLR5-Flagellin 1:1 heterodimer structure, TLR5 ectodomain adopts a single-domain LRR structure that consists of an N-terminal b-hairpin cap (LRRNT) and 13 complete LRR modules (LRR1 to LRR13, not all 25 LRR modules of the zebrafish TLR5 were solved). The concave surface displays a smooth, curved b-sheet structure that is formed from two antiparallel b-strands of LRRNT and 13 parallel b-strands of LRR modules. The convex surface, on the other hand, displays an assortment of helices and extended structures. An ascending lateral surface and a descending lateral surface can also be identified. Of the domains of flagellin, D1 was found to physically interact with TLR5. D1 is mainly composed of three a-helices that bundle together to form a rod. Two of the a-helices are located in the N-terminal portion of D1 (designated αND1a and αND1b, see Fig. 3), and one in the C-terminal portion (designated αCD1). The extensive binding interface of the TLR5-Flagellin 1:1 heterodimer was formed between the ascending lateral surface (LRRNT to LRR10) of TLR5 and one side of the three a-helices of flagellin. Of note, the binding interface of flagellin contains residues involved in flagellin oligomerization and is therefore highly conserved, supporting the hypothesis that TLRs evolved to recognize functionally important structures that are evolutionarily constrained. The binding interface covers a buried surface area (b.s.a.) of ~ 1320 Å^2^ and can be described as two adjacent but separate interfaces A and B. Interface A constitutes ~ 40% of total b.s.a. and is formed through hydrophilic interactions between the concave and ascending lateral surface of TLR5 LRRNT to LRR6 (the N-terminal, beginning portion of TLR5) and the C-terminal half of flagellin αCD1. Interface B constitutes ~ 60% of total b.s.a. and is formed through hydrophilic interactions between the ascending lateral surface of TLR5 LRR7 to LRR10 (the central LRRs) and the upper part of flagellin αND1a and αND1b. Of particular importance in Interface-B is the loop of LRR9; upon flagellin binding, the LRR9 loop undergoes a conformation change to form a well-defined groove, into which the side chain of the conserved flagellin Arg^90^ deeply inserts. This groove alone constitutes ~ 35% of total b.s.a. and includes half of the total H bonds. The importance of this groove interaction was highlighted when an LRR9-loop deletion reduced flagellin binding by at least three orders of magnitude. The Arg^90^-LRR9-loop binding likely forms the hot spot in the TLR5-flagellin interaction [42].

Following the binding of flagellin to TLR5, two TLR5-Flagellin 1:1 heterodimers can then dimerize into a 2:2 tail-to-tail signaling complex [43]. The dimerization interface consists of three surfaces: interface a (TLR5’-flagellin), its two-fold symmetry-related interface a’ (TLR5-flagellin’), and interface b (TLR5-TLR5’) (Fig. 4, top view). Dimerization interface a is formed through hydrophilic interaction between the convex surface of TLR5’ LRR12/13 and the C-terminal ND1b of flagellin. Dimerization interface b is formed through hydrophobic interaction between the ascending lateral surfaces of LRR12/13 of the two TLR5s. The dimerization interface showed high sequence conservation and shape complementarity, indicating functional importance. To determine the significance of dimerization in TLR5 signaling, mutations were introduced into flagellin C-terminal ND1b to disrupt dimerization interface a. Results showed that these mutations severely disrupted TLR5 signaling (by ~ 800-fold) while, interestingly, binding of flagellin to TLR5 was not significantly affected (by only 9-fold). This indicates that for TLR5-flagellin interaction, flagellin binding and 2:2 dimerization can be decoupled (i.e. binding and dimerization are two separate events) and that dimerization is necessary for TLR5 signaling (Fig. 4) [44].

While flagellin D1 is involved in TLR5 binding and dimerization, D0 of flagellin was found to be critical for signaling. Flagellin lacking the D0 domain showed a ~ 1000-fold decrease in TLR5 signaling activity (but no significant change in TLR5 binding) [42]. Further experiments demonstrated that, whereas the N-terminal portion of D0 is expendable for TLR5 signaling, the spoke region (connecting D0 and D1) and hydrophobic residues at the C-terminus of the C-terminal D0 are critical for signaling. Evidence hinted that D0 could be involved in dimerization: flagellin without D0 showed no signaling activity, but when two flagellin molecules lacking D0 domains were covalently cross-linked, signaling activity could be restored. Recent studies also found that D0 is involved in flagellin recognition by the intracellular NAIP5/NLRC4 inflammasome receptor [45]. Through the type 3 secretory system, some pathogenic bacteria can transfer flagellin into the cytoplasm of the host cell as a virulence factor, where it is identified by NAIP5 and NAIP6. Caspase-1 activation occurs by the NOD-like receptor NLRC4 through the interaction of the NLRC4 and NAIP5-flagellin [46]. Previously, it was thought that 35 amino acids from the C-terminus activate the inflammasome, but recent studies found that the N-terminus and the spoke region are also involved in NAIP5-NLRC4 complex formation and caspase-dependent cell death. Tenthorey et al. further showed that the N-terminus makes fewer contacts with NAIP5 while the C-terminal residues 441 to 475 have continuous contact with NAIP5. In summary, evidence indicates that D0 plays a central role in TLR5 dimerization and is the main target for recognition by the intracellular NAIP5/NLRC4 inflammasome receptor [47]. Overall, structural and functional analysis of flagellin thus far concludes that D2 and D3 do not contribute to TLR5 signaling [48].

D1 contains three a-helices that bind to the LRR ectodomain of TLR5, with Arg^90^ on flagellin as the binding hot spot. D0 is required for TLR5 signaling, potentially contributing to dimerization and 2:2 signaling complex formation.

## Application of flagellin as adjuvant with vaccine designs

### Free flagellin: co-administration of flagellin as adjuvant enhances humoral immunity

When co-mixed with vaccine antigens, full-length flagellin monomers have been shown to elicit systemic and mucosal antibody immune responses [49]. Many types of immunocytes and epithelial cells in the mucous tissues express an abundant amount of TLR5 and a strong immune response is observed upon flagellin stimulation [50]. Co-administration of flagellin as an adjuvant works well for many infectious diseases. Inactivated influenza virus with an admix of full-length flagellin resulted in comprehensive humoral response and granulocytes, monocytes/macrophages recruitment to the respiratory tract. Complete protection was observed against a high-dose challenge (but interestingly, no significant enhancement of mucosal IgA was observed as one might expect) [51]. In other studies, humoral immune response and certain degrees of protection can be seen against *Plasmodium vivax* [52], human papillomavirus [53], and foot and mouth disease virus [31]. A simple co-mixture of flagellin with a target antigen may activate innate and adaptive immunity for antigen-specific immune response.

### Genetically linked flagellin: fusion of flagellin to vaccine antigens provides superior adjuvant effect

While the co-administration of flagellin exhibited sufficient adjuvant effect, it soon became clear that physically linking flagellin to vaccine antigens promotes faster and stronger immunogenicity. The working theory behind this observation is that an antigen-PAMP fusion construct provides the two required signals, (1) antigen and (2) PAMPs for costimulatory molecules, simultaneously to the same APC for activation and maturation [17]. In comparison, if antigens and PAMPs were not linked, an APC may only receive one of the signals, resulting in less efficient antigen presentation.

Huleatt and colleagues first demonstrated the importance of antigen-PAMP linkage in a study that linked chicken ovalbumin (OVA) to the C-terminus of full-length flagellin fljb (flagellin phase 2), also called “STF2”, of *S. typhimurium*. Results showed that linking flagellin to the antigen OVA elicited rapid and potent antigen-specific cellular and humoral immune responses comparable to that induced by Complete Freund’s Adjuvant. This boost in immune response required the physical linkage of antigen and flagellin as a simple co-mixture failed to generate an immune response in vivo [54]. To demonstrate improvement of protective efficacy by flagellin linkage, the study performed another experiment linking STF2 to antigens from *Listeria monocytogenes*. Vaccination with the fusion construct stimulated antigen-specific CD8 T cell response and provided significant protection upon bacteria challenge. This immune potentiation by linked flagellin was further demonstrated for other pathogens such as influenza and malaria [52, 55]. On the other hand, linking antigens to the N-terminus of flagellin has also worked for pathogens such as uropathogenic *Escherichia coli* [56], pox virus [57], and a Human Immunodeficiency Virus model [58].

The physical linkage of flagellin to vaccine antigens allows efficient antigen presentation by APCs, reduces effective dosage, and obviates the need for other potentially toxic adjuvants.

### Genetically linked, shortened flagellin: flagellin containing only the conserved D0 and D1 domains retains its TLR5 signaling activity and can act as an adjuvant

Full-length flagellin application as an adjuvant is a potential risk due to its high inflammatory and antigenic properties. At higher doses, it leads to severe systemic inflammatory events which results in liver injury in mice [59]. Removal of the hypervariable region and substitution of it with HIV-p24 not only exuberates IgA antibody release but also reduces the unwanted inflammatory events and antigenicity in mice [60] A detailed comparison of full-length and truncated flagellin is provided in Table 1.

**Table 1:** Comparison of full-length and truncated flagellin studies

Features	Full-Length Flagellin (e.g., FljB, FliC)	Truncated Flagellin (e.g., Hag, tmFliC)	References
Domains	D0, D1, D2, D3	D0, D1 (lacks D2, D3)	[82, 83]
TLR5 Activation	Strong activation, robust NF-κB signaling	Moderate activation, lower TLR5 signaling potency	
Proinflammatory Response	High IL-6 and TNF-α levels, causing inflammation	Reduced IL-6 and TNF-α, lower inflammation	
Immune Response (Adjuvant)	Strong antibody production and cellular immune response	Effective but milder immune response, safer with fewer side effects	
Purification Efficiency	Low due to polymeric filament formation	High, monomeric form improves solubility and yield	
Clinical Potential	Effective but may require modification to reduce adverse effects	Lower risk of side effects, promising for safer repeated use	
Cost Efficiency	Higher cost due to complex purification	Lower cost, easier to purify	

Moreover, structural and functional studies of flagellin have concluded that D0 and D1 are required for TLR5 activation while the D2 and D3 hypervariable domains are dispensable due to the following reasons [61]. Initially, it triggers a pronounced cytokine storm, leading to significant inflammation and localized tissue damage. Subsequently, the hypervariable regions (D2 and D3) are instrumental in the generation of neutralizing antibodies against flagellin [62]. Finally, extensive mutation and structural studies reveal that these regions have an inconsequential role in TLR5 binding and signal transduction. Therefore, a further advancement in flagellin adjuvant design employs a ‘shortened flagellin (D0 + D1)’ instead of the full length. The reduction in flagellin molecular weight by half results in smaller fusion constructs, which could (1) allow easier protein refolding of the antigen and flagellin, and (2) increase protein expression quantity. Also, without the hypervariable region, less antibody response will be generated against flagellin [63]. The bulk of the reports on using flagellin as an adjuvant take this approach of linking antigens to the shortened flagellin at the N-, C-terminus, or in place of the hypervariable region. A recent study on SARS-CoV-2 variants uses all three sites of flagellin to attach the receptor binding domain (RBD) of Spike protein. The study revealed that the resulting RBD-flagellin vaccine induced enduring and broad-neutralizing IgG in plasma as well as IgA in salivary secretions [64]. Truncated flagellin retains structural, thermal, and functional stability when key TLR5-interacting regions are preserved. Circular dichroism confirmed secondary structure stability, while thermal assays demonstrated heat resilience. Functional stability was validated through TLR5 activation in Caco-CCL20-LUC reporter cells. These findings indicate that truncation does not significantly affect the protein’s ability to activate TLR5, provided its essential interaction domains are maintained [48].

## D0 and D1 domains from bacteria: a new frontier in adjuvant technology

Structural analysis of flagellin across different bacterial species has identified variants with four, three, and two domains. The flagellin structures of *B. subtilis*, *B. cereus*, and *Treponema pallidum* have been revealed to consist of these conserved domains (D0, D1). Previously, studies on *S. typhimurium* flagellin, which comprises four domains, often involved truncating the hypervariable domain [62]. With the advent of these conserved domain flagellins, antigens can be simply attached to either the N or C-terminus of these domains. Studies of *B. subtilis* flagellin have demonstrated its potent immunogenic nature with less reactogenicity compared to *S. typhimurium* when applied with the matrix 2 protein (M2e) of the influenza virus [65]. In addition to N or C-terminal attachment, there is the possibility of antigen insertion at *B. cereus* Flg residues 178–180, as determined by cellular and biophysical analyses. This insertion of foreign antigens does not interfere with TLR5 activation [66].

## Quest for simplicity: deconstructing the shortened flagellin further for minimal flagellin epitopes required for TLR5 activation

Flagellin activates TLR5 and cytosolic NOD-like receptor (NLR) protein 4 (NLRC4) receptors to orchestrate adaptive immune responses against specific pathogens. However, mutation studies revealed that flagellin lacking NLRC4 activation raised significantly higher antibody titers compared with wild-type flagellin. It implies that TLR5 activation is downregulated by the NLRC4 activation. Li et al. concluded that TLR5 is the principal pathway to contribute to flagellin-derived immune stimulation [67]. Considering their findings, we design a truncated flagellin 1-99aa of flagellin incorporating hot-spot region R89. Linking of truncated flagellin to antigens results in significantly higher antibody titers, cytokines release, and protection during challenge in the case of Pasteurella *multocida* [68], *Duck Hepatitis* A Virus Serotype 3 [69], and *Actinobacillus pleuropneumoniae* [70]. A subunit foot-and-mouth disease virus vaccine was developed incorporating self-assembling nanoparticles (i301), VP1, and flagellin 1–99. The nano-flagellin component elicited a robust increase in antigen-specific neutralizing antibody responses and provided superior protection in challenge assays conducted on guinea pigs [71]. In short, our work supports the notion of TLR5 as the principal pathway of flagellin adjuvancy. Recently, one more study reported the use of D1 as an inducer in pro-inflammatory cytokines releases up to multi-fold for TNF-α, Il-8, and IL-6. However, the study lacks details about TLR5 activation and adjuvant use of D1 [72]. In another approach, a small peptide from flagellin (residues 85–111, named pFlg) containing the critical TLR5 binding hot spot was engrafted onto liposomes to deliver tumor antigens or DNA to DCs. Direct binding of the liposomes to the DCs was observed and the binding was correlated with TLR5 expression, indicating that the pFlg on liposomes can help target DCs by binding to TLR5. Augmented immune response was observed because of the DC-targeting [73]. Later, another study took a similar approach by linking pFlg to the C-terminus of the porcine circovirus 2 (PCV2) capsid protein, which self-assembles into virus-like particles (VLPs). Enhanced delivery of the PCV2 VLPs to DCs was hypothesized. Vaccination resulted in better humoral and cellular immune response and lower viremia after challenge [72]. These studies demonstrated that pFlg can help boost immune response, presumably by binding to TLR5 on DCs to deliver the antigens. It remains to be demonstrated directly, however, that pFlg can activate TLR5 signaling. It is conceivable that pFlg alone is sufficient for TLR5 binding since a later structural study showed that the binding hot spot is indeed contained within pFlg. However, it comes as a surprise that without any parts of D0, pFlg may activate TLR5 signaling. Pre-clinical studies on flagellin are summarized in Table 2. Future investigation is warranted.

**Table 2:** Pre-clinical studies on flagellin as vaccine adjuvant

Flagellin Origin	Disease Model	Animal Used	Immune Response	References
*S. typhimurium*	Human Influenza	Mice	Strong humoral response, and protection against lethal challenges in mice.	[55]
*S. typhimurium*	Malaria (*Plasmodium vivax*)	Mice	Induction of systemic and mucosal antibodies, TLR5-mediated cytokine release enhancing immune protection.	[52]
*V. vulnificus*	Tetanus Toxoid (TT)	Mice	Elevated mucosal immunity with IgA production, protecting against challenge and establishing systemic immunity.	[29]
*P. multocida*	Avian Pasteurellosis	Chicken	Increased antibody titers, cytokine release, and improved survival rate upon challenge infection.	[68]
*B. subtilis*	Influenza	Mice	Enhanced immunogenicity with reduced pro-inflammatory effects, high mucosal IgA response.	[82]
*S. typhimurium*	Foot-and-Mouth Disease	Guinea Pigs	Increased neutralizing antibodies, providing superior protection through a self-assembling nanovaccine incorporating flagellin.	[71]
*E. coli*	Genital cancer	Mice	Enhanced vaginal immune responses with adjuvanted therapeutic vaccine leading to potent immune activation.	[53]
*S. typhimurium*	Infectious Bursal Disease Virus (IBDV VP2)	Chicken	Enhanced cytokine expression (IL-4, IFN-γ), potentiate IgG release and virus neutralizing antibodies.	[33]
*S. typhimurium*	Porcine epidemic diarrhea virus (PEDV)	Mice	Elicit higher IgG titers, T-cell response and higher titers for neutralizing antibodies.	[84]

## Flagellin: a potential hapten carrier in addition to its established adjuvant role

Flagellin serves a dual purpose in vaccine development, functioning as both an adjuvant and a hapten carrier. As an adjuvant, flagellin activates Toll-like receptor 5 (TLR5), stimulating innate immune responses and enhancing immunity against co-administered antigens, particularly for mucosal immunization. As a hapten carrier, flagellin can bind to small, non-immunogenic molecules (haptens), enabling the immune system to produce hapten-specific antibodies. For example, in one study, a cocaine hapten called GRE was fused to flagellin and administered to mice, where the flagellin-GRE construct elicited higher levels of anti-GRE antibodies than other groups, demonstrating flagellin’s potential in hapten recognition [75]. Building on this, a lipid-A-free lipopolysaccharide was linked to flagellin to create a conjugate vaccine, which generated a targeted immune response, resulting in 80% survival 28 days post-challenge [76]. These studies suggest that flagellin can effectively elicit antigen- and hapten-specific immune responses, though further research is needed to fully understand hapten-flagellin interactions [73, 74].

### Flagellin established safety in clinical trials

The therapeutic application of drugs in real-world problems is the goal of drug development studies. In the case of flagellin, VaxInnate Corporation sponsored an influenza vaccine VAX102 comprised of *S. typhimurium* flagellin type 2 (STF2; TLR5 ligand) fused to influenza A virus protein M2e. The vaccine went to Phase 1 trial with 60 participants and it induced antibody titers to M2e with mild to moderate issues related to safety. All immune responses were in the safety window with no adverse events [75].

More recently, a SARS CoV-2 vaccine was developed combining *E. coli* flagellin and RBD domains of spike protein. The subunit vaccine was tested in mice followed by a clinical study on human volunteers. Broad-neutralizing IgG and IgA titers were measured both in mice and humans with favorable safety profiles [62].

Mobilan, an anticancer gene therapy, was established using an adenovirus vector carrying the genes for TLR5 and flagellin. Mice responded well to Mobilan treatment, showing immune activation, a decrease in metastasis, and a decline in tumor growth. Mobilan was injected into the human prostate, and it induced self-resolving inflammation and immune cell infiltration in prostate tissue. Moreover, Mobilan was safe and well-tolerated at all doses [76].

A derivative of *Salmonella* flagellin, Entolimod, has been tested for its potential antiradiation and anticancer therapy [37]. However, Entolimod was rapidly neutralized by preexisting antibodies during Phase 1 human studies. Later, a modified drug (GP532) was designed by removing B-cell, T-cell and inflammasome-activating domains. GP532 exhibited a potent release of cytokines which protect mice in anti-radiation therapy. Moreover, it showed better suitability for multidose therapy for patients with preexisting neutralizing antibodies [77]. Clinical uses of flagellins are presented in (Table 3).

**Table 3:** Clinical study on flagellin as therapeutic agent

Flagellin Origin	Disease Model	Study Subjects	Immune Response	References
S. typhimurium	Influenza Virus (VAX102)	Human Volunteers	Induced antibody titers to influenza virus with moderate adverse safety concerns.	[77, 80]
S. typhimurium fljB	Influenza virus (HA) antigen, VAX125)	Mice and Human	Safe and efficacious TLR mediated-immune response in elderly individuals against seasonal influenza infection	[85, 86]
S. typhimurium	Anti-radiation therapy	Human Volunteers	Strong initial cytokine response, but neutralizing antibodies reduced Entolimod’s efficacy in humans; modified GP532 showed effective cytokine release and protection in mice	[37, 79]
S. typhimurium	Cancer Immunotherapy	Human Prostate Cancer Patients	Induced local inflammation and immune cell infiltration in prostate tissue without severe adverse effects, showing safety and immune activation.	[78]
E. coli	SARS-CoV-2	Mice and Human	Broad neutralizing IgG and IgA titers both systemically and in mucosal tissues, with favorable safety across human and animal subjects.	[64, 82]

## Challenges in the clinical trials and market translation of flagellin-based therapies

Flagellin’s advancement through clinical trials and toward commercialization has been constrained by reactogenicity and the induction of neutralizing antibodies. In early Phase 1 studies with seasonal influenza vaccines, flagellin was tested as an adjuvant in VAX102 (STF 2.4×M2e), created by fusing flagellin’s C-terminus with four tandem repeats of the M2e matrix protein and co-administered with a trivalent inactivated influenza vaccine (TIV). These trials reported mild to moderate side effects, such as headache, fatigue, and muscle pain, but overall tolerability was good [77]. In a separate Phase 1 study, the STF 2.4×M2e subunit alone led to severe adverse effects at higher doses (3 µg and 10 µg), attributed to elevated cytokine levels and C-reactive proteins [80]. Later, a quadrivalent influenza vaccine with VAX102 elicited severe symptoms in a few participants at higher doses, although it was generally well-tolerated [81]. However, the lack of progression of these vaccines into subsequent clinical studies could be attributed to minor to moderate clinical events observed within the targeted population.

Flagellin trials for its use as a radioprotective agent (Entolimod), flagellin’s effectiveness was reduced by neutralizing antibodies, which compromised its efficacy as radioprotective agent. To address this, a modified form, GP532, was developed, showing resistance to these antibodies in murine models [37]. Overall, the risks of cytokine release and antibody-mediated responses against flagellin have posed barriers to flagellin’s clinical progress to market.

### Flagellin-TLR5 hot-spot binding region is pivotal to flagellin-based adjuvancy and future flagellin truncation designs

The shortened flagellin represents the most effective form of flagellin, with the hot-spot region playing a pivotal role in any flagellin-based adjuvant design. Numerous studies have confirmed that mutations in this hot-spot region disrupt TLR5 activation [78]. To pinpoint the minimal flagellin epitope required for TLR5 binding, a range of constructs incorporating residues from D0 and D1, including the essential hot-spot region, should be tested. Specifically, engineering the spoke region and the hydrophobic tail of the C-terminal D0 may enhance effective signaling. The final optimized form of flagellin holds great promise for future subunit vaccine designs.

## Conclusions

The flagellin as a vaccine adjuvant highlights its substantial potential to enhance immune responses through TLR5 activation. While significant progress has been made by structural biology in identifying the critical domains responsible for its adjuvant activity, the structural complexity of flagellin continues to present challenges in optimizing its use. The focus on the D0 and D1 domains has unfolded avenues for developing streamlined and effective antigen-adjuvant constructs, yet further research is mandatory to refine these domains to their minimal functional units. Additionally, investigating the application of flagellin in various clinical contexts, such as its role in cancer immunotherapy, anti-radiation, and as a hapten carrier, may unlock new therapeutic potential. As flagellin continues to advance in clinical studies, its multifunctional capabilities as an adjuvant and beyond hold flair for the development of next-generation vaccines and therapeutics.

## Data Availability

No datasets were generated or analysed during the current study.
